# Diversion of phagosome trafficking by pathogenic Rhodococcus equi depends on mycolic acid chain length

**DOI:** 10.1111/cmi.12050

**Published:** 2012-11-13

**Authors:** Tobias Sydor, Kristine Bargen, Fong-Fu Hsu, Gitta Huth, Otto Holst, Jens Wohlmann, Ulrike Becken, Tobias Dykstra, Kristina Söhl, Buko Lindner, John F Prescott, Ulrich E Schaible, Olaf Utermöhlen, Albert Haas

**Affiliations:** 1 Institute for Cell Biology, University of BonnBonn, Germany; 2 Department of Medicine, Washington UniversitySt. Louis, MO, USA; 3 Research Centre BorstelBorstel, Germany; 4 Department of Pathobiology, University of GuelphGuelph, ON, Canada; 5 Department of Medical Microbiology, Immunology and Hygiene, University of CologneCologne, Germany; 6 Center for Molecular Medicine Cologne, University of CologneCologne, Germany

## Abstract

*Rhodococcus equi* is a close relative of *Mycobacterium* spp. and a facultative intracellular pathogen which arrests phagosome maturation in macrophages before the late endocytic stage. We have screened a transposon mutant library of *R. equi* for mutants with decreased capability to prevent phagolysosome formation. This screen yielded a mutant in the gene for β-ketoacyl-(acyl carrier protein)-synthase A (KasA), a key enzyme of the long-chain mycolic acid synthesizing FAS-II system. The longest *kasA* mutant mycolic acid chains were 10 carbon units shorter than those of wild-type bacteria. Coating of non-pathogenic *E. coli* with purified wild-type trehalose dimycolate reduced phagolysosome formation substantially which was not the case with shorter *kasA* mutant-derived trehalose dimycolate. The mutant was moderately attenuated in macrophages and in a mouse infection model, but was fully cytotoxic.Whereas loss of KasA is lethal in mycobacteria, *R. equi* *kasA* mutant multiplication in broth was normal proving that long-chain mycolic acid compounds are not necessarily required for cellular integrity and viability of the bacteria that typically produce them. This study demonstrates a central role of mycolic acid chain length in diversion of trafficking by *R. equi*.

## Introduction

*Rhodococcus equi* is a nocardioform Gram-positive coccobacillus that can cause severe pyogranulomatous pneumonia in young horses and tuberculosis-like symptoms and histopathology in AIDS patients. *R. equi* is a facultative intracellular bacterium, based on its capability to survive and multiply in macrophages *in vitro* and *in vivo* (Hietala and Ardans, 1987; Zink *et al*., 1987; Hondalus and Mosser, 1994). Virulence for foals and mice strictly depends on the presence of a ∼ 85 kbp virulence-associated plasmid (VAP) (Takai *et al*., 1991). In macrophages, phagosomes containing virulent (VAP-positive) *R. equi* are arrested early in their maturation and acquire neither the proton-pumping vacuolar ATPase complex nor lysosomal hydrolases (Fernandez-Mora *et al*., 2005). After 1–3 days of infection, macrophages die by necrosis, at which time intracellular multiplication has occurred (Lührmann *et al*., 2004). In contrast, avirulent plasmid-cured *R. equi* are delivered into acidified phagosomes which, eventually, mature into phagolysosomes (Fernandez-Mora *et al*., 2005) where bacteria are slowly killed (von Bargen *et al*., 2009). Plasmid-less bacteria have reduced cytotoxic effect on their host macrophages (Lührmann *et al*., 2004).

**Fig 1:**
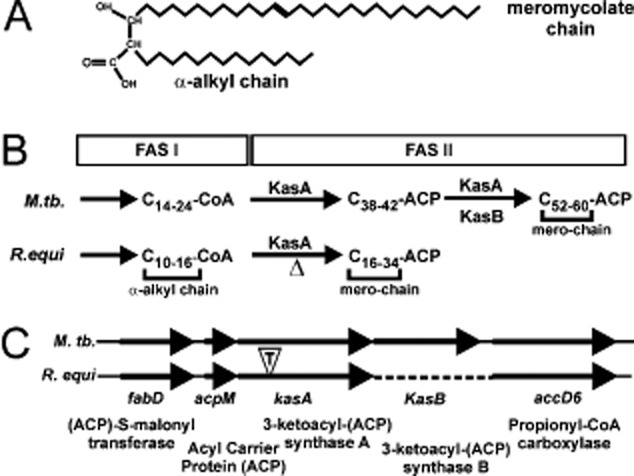
Organization of Kas genes and processing functions of mycolic acid precursors.A. Structure of a typical mycolic acid from *Rhodococcus equi*.B. The mycobacterial FAS-I enzyme system generates C_14_–C_24_ fatty acids which are either used directly or are transferred to the KasA-dependent FAS-II system for extension by two carbon units per enzymatic cycle up to 42 carbon units. Further extension of long KasA-dependent mero-segments to very long ones (up to C_60_) is further dependent on KasB. KasB also participates in cyclopropanation in mycobacteria, a modification absent from *R. equi* mycolic acids. *R. equi* FAS-I produces C_10_–C_16_ fatty acids which are elongated up to 34 carbon units by FAS-II. The resulting FAS-II-derived fatty acids are incorporated into mycolic acids as their mero-segments and the FAS-I-derived C_12_–C_18_ as α-alkyl chains. ‘Δ’ denotes the missing enzymatic activity in the *R. equi* *kasA* mutant. Scheme based on Slayden and Barry (2002), Gao *et al*. (2003), Brown *et al*. (2005), Takayama *et al*. (2005) and Hsu *et al*. (2011b).C. Organization of the *kasA* gene regions on the *M. tuberculosis* and *R. equi* chromosomes is identical but *R. equi* lacks a *kasB* gene (dotted line). The insertion position of the transposon in mutant FA11 is denoted by a ‘T’ in an open triangle. The open reading frames in *R. equi* are deposited as YP_004007593 (FabD), YP_004007594 (AcpM), JN_030359 (KasA) and YP_004007596 (AccD6).

The genera *Rhodococcus*, *Mycobacterium*, *Corynebacterium* and *Nocardia* belong to the actinomycetes group and harbour several pathogenic species. One of their hallmarks is the possession of mycolic acids, i.e. α-alkylated β-hydroxylated fatty acids with a mostly constant-length short α-alkyl and a meromycolate side chain of up to 60 carbon units in length (for rhodococcal mycolic acid structure, see Fig. 1A). To produce long-chain mycolic acids, a monomeric fatty acid synthase I (FAS-I) system first generates short α-alkyl chains which are transferred to a multimeric fatty acid synthase II (FAS-II) system for elongation by two carbon units per enzymatic cycle (Fig. 1B). Components of the FAS-II system are targets of some anti-tuberculosis drugs such as isoniazid and thiolactomycin. Isoniazid is a prodrug which is activated by the mycobacterial catalase–peroxidase KatG and which blocks InhA (2-*trans* enoyl-acyl carrier protein reductase) activity. Thiolactomycin inhibits mycobacterial β-ketoacyl-(acyl carrier protein)-synthase A (KasA) and B (KasB), carbon–carbon condensing enzymes of the FAS-II system. In mycobacteria, KasA is responsible for long-chain production and indispensable for growth (Bhatt *et al*., 2005) whereas mycobacterial KasB adds a few of the final carbon units to the growing chains and is likely involved in mycolic acid modification by mycobacteria (Bhatt *et al*., 2007a). Gao *et al*. showed that the *Mycobacterium marinum kasB* gene can be deleted leading to 2–4 carbon units shortened mycolic acids with an overall length of 79–85 carbon units in wild-type cells versus 77–81 in *kasB* mutants (Gao *et al*., 2003). The chromosomal organization of the *kasAB* region is similar in *Mycobacterium tuberculosis* and *R. equi* (Fig. 1C).

Corynebacteria have no functional FAS-II system at all, but condense two FAS-I product size fatty acids to form short 22–36 carbon unit mycolic acids (Barry *et al*., 1998; Sacco *et al*., 2007). Rhodococcal mycolic acids are 34–48 carbon, nocardial ones 44–60 carbon units in size and pathogenic mycobacteria, such as *M. tuberculosis*, produce the longest chains (up to C_64_ meromycolates with a C_26_ α-chain). Long-chain mycolic acid compounds are largely responsible for the wax-like character of the actinomycete cell wall and the formation of a mycobacterial ‘outer membrane’ (Liu *et al*., 1995; Hoffmann *et al*., 2008). A portion of them is incorporated into glycolipids, in particular trehalose dimycolate (TDM), which is a granulomagenic, immunostimulatory and toxic lipid of the cell wall (Rhoades *et al*., 2003; Bhatt *et al*., 2007b; Matsunaga and Moody, 2009; Harris *et al*., 2010). Another portion is covalently linked into the mycolic acid–arabinogalactan–peptidoglycan of the cell wall. Chain length variation and the degree of saturation are the only modifications found in rhodococcal mycolic acids (Sutcliffe, 1998; Hsu *et al*., 2011a) whereas mycobacterial mycolic acids can contain methyl-, cyclopropyl-, methoxy- and keto-group modifications (Barry *et al*., 1998).

Several biochemical reports suggested a role of complex glycolipids in deviation of phagosome biogenesis by pathogenic *Mycobacterium* species (Spargo *et al*., 1991; Fratti *et al*., 2003; Gao *et al*., 2003; Indrigo *et al*., 2003; Vergne *et al*., 2004; Robinson *et al*., 2007; Axelrod *et al*., 2008; Brodin *et al*., 2010; Neyrolles and Guilhot, 2011). By analogy, complex surface glycolipids, such as TDM (or ‘cord factor’) and lipoarabinomannan (Garton *et al*., 2002; Niescher *et al*., 2006), are regarded as putative rhodococcal virulence-associated factors, although there is little experimental evidence and only inferred from genetically heterogeneous isolates (Gotoh *et al*., 1991). Since non-pathogenic actinomycetes can also possess very-long-chain mycolic acids (Barry *et al*., 1998), modulation of phagosome maturation is likely not their only purpose. Accordingly, alterations in mycolic acid biosynthesis come with profound physiological and morphological defects (Takayama *et al*., 2005). Non-virulence roles for mycolic acids, mostly inferred from mutant analysis, include resistance to antibiotics, ultraviolet and γ-irradiation, oxidative stress, various chemicals, desiccation and regulation of biofilm formation (Brennan and Nikaido, 1995; Ojha *et al*., 2005).

Here, we present direct genetic, biochemical and cell biological evidence that long-chain mycolic acids are not required for growth of *R. equi* in rich or minimal media, but are key players in diversion of phagosome trafficking by this pathogen.

## Results and discussion

### Functional screening of *R. equi* phagosome trafficking mutants

To identify *R. equi* factors that are involved in diversion of phagolysosome formation, we adapted an established protocol (Pethe *et al*., 2004). Briefly, in this protocol macrophage cell lysosomes are pre-labelled by a paramagnetic compound and these cells are subsequently infected with the pathogen. After sufficient time for phagolysosome formation to occur, macrophages are homogenized, (phago-)lysosomes are collected with a magnet and contained pathogen mutants are grown on nutrient broth. Bacterial mutants selected in this way are used to re-infect macrophages. Repetition of this procedure should yield mutants that are generally more frequently delivered to phagolysosomes.

Here, a mutant bacteria library was added to J774E macrophage-like cells whose lysosomes had been pre-loaded with 10 nm paramagnetic particles. After a 2 h chase, macrophages were lysed; magnetic (phago-)lysosome fractions were separated and spread on nutrient agar. Clones of three independent mutants were enriched by seven rounds of selection and the mutant which was most frequently obtained (FA11) was further investigated.

### The mutated gene in FA11 encodes a β-ketoacyl-(acyl carrier protein)-synthase

To identify the insertion locus in the *R. equi* genome, we cloned the transposon with neighbouring DNA sequences from FA11 in *E. coli* and determined their nucleotide sequences as described (Sydor *et al*., 2008). Transposon-neighbouring sequences were analysed using the blast program offered by the *R. equi* genome sequencing web page of the Wellcome Trust Sanger Institute. The nucleotide sequences were identical to nucleotides 3 076 584 to 3 077 831 (now GenBank Accession Number JN_030359; Letek *et al*., 2010). Insertion of the transposon in FA11 had occurred in an open reading frame coding for a 415-amino-acid sequence. Comparison with all deposited bacterial protein sequences using the National Center for Biological Information blast program identified the mutated gene as a close homologue of mycobacterial *kasA* and *kasB* genes which code for β-ketoacyl-acyl carrier protein (ACP) synthases of the FAS-II complex (Fig. 1B and C). *R. equi* KasA was 67.4% identical and 82.6% similar to *M. tuberculosis* KasA (Fig. S1) and 61.9% identical to *M. tuberculosis* KasB. Hence, *R. equi* Kas is, by amino acid sequence criteria, as much related with *M. tuberculosis* KasA and KasB as these are with each other (66% identity; Takayama *et al*., 2005). The amino acid residues of the KasA catalytic triad (in *M. tuberculosis*, Cys^171^, His^311^, His^345^; Kremer *et al*., 2000) were present at equivalent positions in the *R. equi* protein (Fig. S1). Whereas *M. tuberculosis* and other mycobacteria possess *kasA* and *kasB* genes organized in an operon, *R. equi* possesses only one *kasA/B* gene (Fig. 1C). This property is shared with *Rhodococcus jostii* RHA1 (McLeod *et al*., 2006), *Rhodococcus erythropolis* (Mitani *et al*., 2006) and *Nocardia farcinica* (Ishikawa *et al*., 2004). We termed the gene identified in this screen *R. equi kasA* following its initial annotation (Letek *et al*., 2010), although our annotation (GenBank Access JN_030359) yielded a KasA version with additional 27 amino acids at its amino terminus (Fig. S1). The transposon is inserted into *kasA* within the codon 134 of a total 415 codons and, hence, after approximately one-quarter of the open reading frame.

Throughout this work, wild-type *R. equi* containing the endogenous virulence plasmid (VAP) will be denoted 103+, a VAP-cured derivative 103− and the VAP-containing *kasA* mutant (FA11) 103+/*kasA*.

### Analysis of 103*+*/*kasA* mycolic acid composition and cell wall structure

KasA and KasB are part of the FAS-II machinery which elongates fatty acids produced by FAS-I complex from C_16_–C_26_ to C_52_–C_64_ in *M. tuberculosis*, or, in rhodococci, up to 34 carbon units with 0–4 double bonds (Sutcliffe, 1998; Takayama *et al*., 2005; Bhatt *et al*., 2007b) (Fig. 1B). The biological functions of such long-chain mycolic acids are unclear because mutants in several aspects of mycolic acid biosynthesis are non-viable or defective in physiological parameters, as shown in a number of landmark contributions (Liu and Nikaido, 1999; Gao *et al*., 2003; Portevin *et al*., 2004; Bhatt *et al*., 2005; 2007a; Barkan *et al*., 2009). Therefore, it is not clear whether observed phenotypes were direct consequences of the respective mutation.

**Fig 2:**
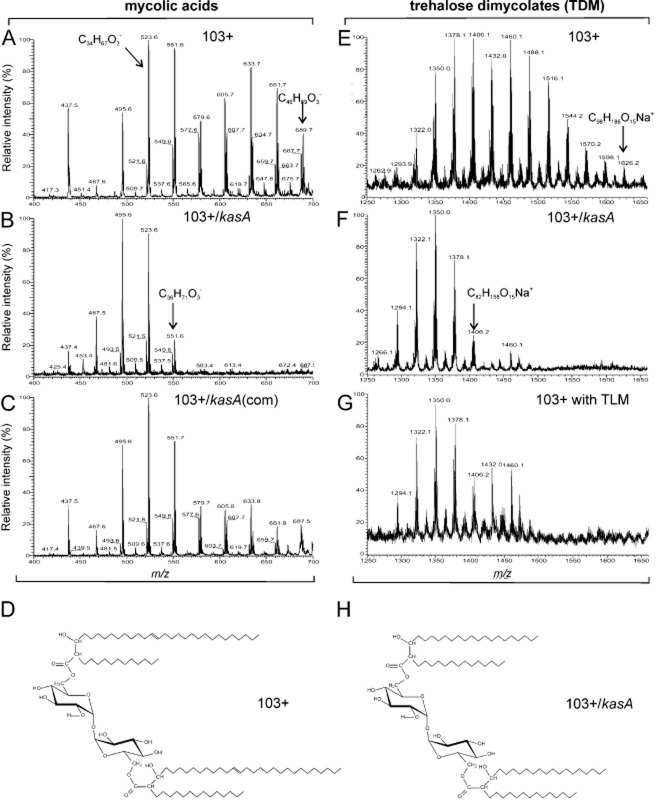
Mass spectrometry of mycolic acids from wild-type and *kasA* mutant cells. Chloroform : methanol extracts of *R. equi* were analysed using LIT ESI-MS (*Experimental procedures*). The *m/z* region of mycolic acids extracted from (A) 103+, from (B) 103+/*kasA* and from (C) 103+/*kasA* complemented with wild-type *kasA* are shown as well as the mass spectra of TDM from 103+ (E), from 103+/*kasA* (F) and from 103+ grown in the presence of 0.2 mg ml^−1^ thiolactomycin (G). The elemental formulas are indicated for some of the compounds. (D and H) Structures of the longest TDM variant from 103+ ([M+Na]^+^ ion = C_98_H_186_O_15_Na^+^, 1626.3 Da) and from 103+/*kasA* ([M+Na]^+^ ion = C_82_H_158_O_15_Na^+^, 1406.1 Da) respectively. Double bond positions are exemplary (Hsu *et al*., 2011a).

One possible effect of *kasA* deletion in *R. equi* could be that bacteria produce mycolic acids with two FAS-I product size acyl chains, as found in corynebacteria which naturally lack FAS-II (Takayama *et al*., 2005). In line with this, mass spectrometry analysis of chloroform : methanol extracted lipids revealed that the maximum chain length of mycolic acids from 103+ was 46 (predominant species: 34–36) carbon units whereas that of 103+/*kasA* was 36 (predominant species: 32–34) carbon units, i.e. 10 carbon units shorter than the corresponding two condensed FAS-I and FAS-II product chains (Fig. 2A and B). This difference was reflected in the molecular mass of the major releasable mycolic acid-containing glycolipid, TDM, with the longest mycolic acid chains comprising 70 carbon units in the *kasA* mutant versus 88 in wild-type cells (Fig. 2D, E, F and H). The chain length difference between wild-type *M. marinum* and its *kasB* mutant mycolic acid is only two to four carbon units, likely because KasB is only a secondary elongation enzyme, acting on chains already elongated by the KasA complex (Gao *et al*., 2003).

Mutant 103+/*kasA* was functionally complemented with the wild-type *kasA* gene expressed from a constitutive mycobacterial *hsp60* promoter although the relative abundance of long chains was lower in the complemented mutant than in wild-type cells (Fig. 2A–C), suggesting that the KasA expression level was lower in reconstituted mutant bacteria.

### Morphology and resistance of 103*+*/*kasA* to antibiotics and FAS-II inhibitors

**Fig 3:**
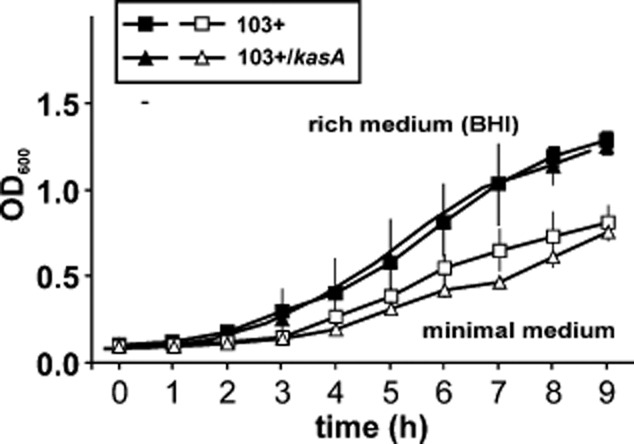
Growth of 103+ and 103+/*kasA* mutant in broth culture. Cultivation of *R. equi* 103+ or 103+/*kasA* in rich BHI broth (closed symbols) or in minimal media containing acetate as the sole carbon source (open symbols) at 37°C for the indicated times. There are no statistically significant growth differences between wild-type and mutant cultures. Cell numbers were quantified as the absorbance at 600 nm. Data are means and standard deviations from three independent determinations.

The finding that 103+/*kasA* was viable at all was surprising since in *M. tuberculosis*, deletion of *kasA* is lethal (Kremer *et al*., 2000; Bhatt *et al*., 2005) although a Kas activity (KasB) exists in all sequenced mycobacterial genomes. KasB is dispensable, but mutant cells have a more permeable cell wall and are more frequently delivered to phagolysosomes (Liu and Nikaido, 1999; Gao *et al*., 2003; Bhatt *et al*., 2007b). Surprisingly, *R. equi* grew normal even in the absence of KasA activity both in rich brain heart infusion (BHI) broth and in minimal media with acetate as sole carbon source (Fig. 3). Yet, KasA is obviously the only Kas activity in the *R. equi* FAS-II system, also because its inactivation completely abrogated production of FAS-II size mycolic acids (Fig. 2G).

**Fig 4:**
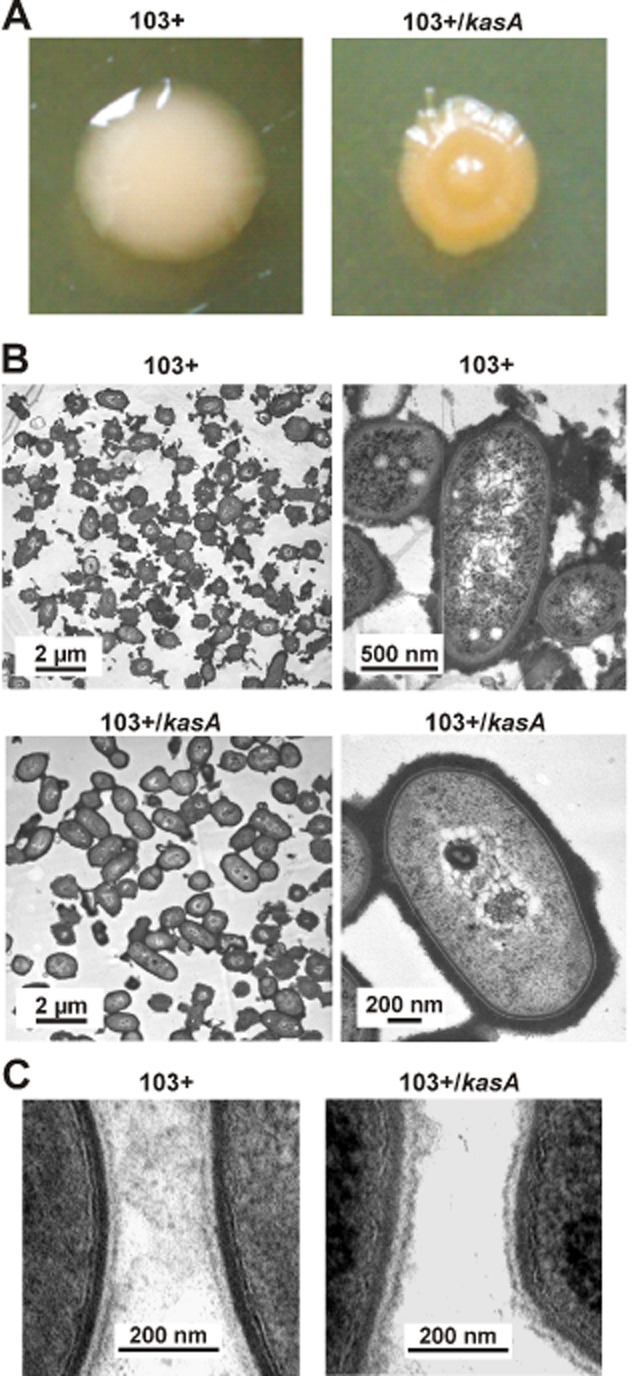
Morphology of *kasA* mutant cells.A. Morphology and colour of single colonies on BHI agar plates after growth at 37°C.B and C. Transmission electron micrographs of wild-type 103+ and 103+/*kasA* mutant cells, in C at very high magnification. The dark fuzzy layer around the bacteria, particularly in B, is the ruthenium red-stained polysaccharide capsule. The cell wall portions of two adjacent bacterial cells are shown in each micrograph in C. Size standards are indicated in B and C.

Strain 103+ colonies were smooth and slimy whereas 103+/*kasA* produced single colonies of smaller size of more dome-shaped and less mucous morphology (Fig. 4A). One of the possible causes for such altered colony surface was loss of the polysaccharide capsule (Sydor *et al*., 2008; von Bargen and Haas, 2009). The capsule of the mutant, as studied by transmission electron microscopy, was similar to that of wild-type cells and somewhat more evenly and densely distributed over the surface (Fig. 4B), possibly explaining the ‘drier’ phenotype. In general, no gross cell wall structure differences were observed between wild-type and mutant bacteria using transmission electron microscopy, although the mutant’s cell wall tended to be thicker (Fig. 4B and C). Additionally, resistance to any of 16 antibiotics in the mutant was little affected, although there was an increased sensitivity to amikacin and streptomycin (Fig. S2) which are hydrophilic. In mycobacteria, changes in mycolic acid composition lead to strongly altered sensitivities particularly to hydrophobic antibiotics (Liu and Nikaido, 1999; Gao *et al*., 2003; Bhatt *et al*., 2007a).

**Fig 5:**
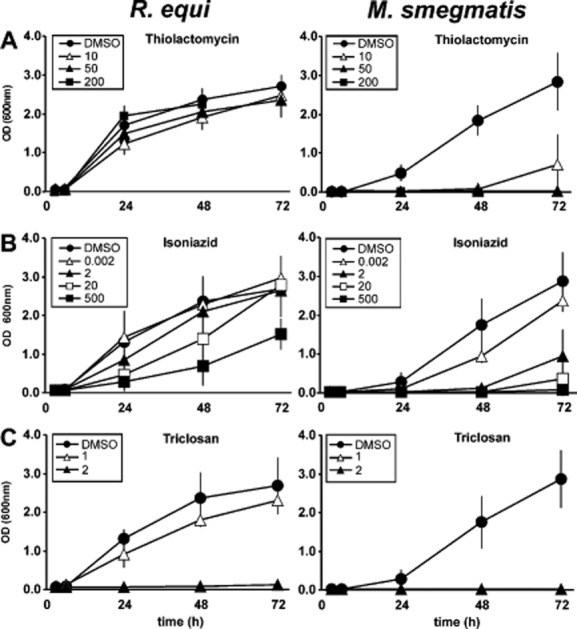
Sensitivity of the *R. equi* and *M. smegmatis* to FAS inhibitors. Bacteria were grown in rich media (LB for *Mycobacterium smegmatis*, BHI for *R. equi*) in presence of the indicated concentrations (μg ml^−1^) of (A) thiolactomycin, (B) isoniazid or (C) triclosan. DMSO carrier controls contained the DMSO amounts corresponding to the 10 μg ml^−1^ drug samples. At the indicated times, samples were removed and absorbance at 600 nm was recorded. Data are presented as means and standard deviations from 3–6 independent experiments.

Several antibiotics have been developed against the mycobacterial FAS-II system, some of them being tested or used against mycobacterial infections. Thiolactomycin (TLM) is a specific inhibitor of enzymes which catalyse a condensation between acyl-ACP and malonyl-ACP with a strong preference for mycobacterial KasA and KasB over the corresponding FAS-I activity (Slayden *et al*., 1996). *Rhodococcus* growth was unaltered by TLM at a high concentration of 200 μg TLM ml^−1^ (Fig. 5), although mass spectrometry revealed the inactivation of its FAS-II system by TLM (Fig. 2G) and although even 50 μg TLM ml^−1^ inhibit growth of *E. coli* by 90% which does not even produce mycolic acids (Hayashi *et al*., 1983). Together with the uninhibited growth of the *kasA* mutant, this indicated that *R. equi* FAS-II is dispensable in BHI broth. Growth of *Mycobacterium smegmatis*, on the other hand, was completely stopped by 10 μg ml^−1^ TLM, the lowest concentration tested (Fig. 5A). *M. smegmatis* was also very sensitive to isoniazid (INH), the paradigm mycobacterial FAS-II system prodrug targeted to the acyl-ACP enolase InhA. INH strongly reduced growth at 2 μg ml^−1^, whereas even 500 μg ml^−1^ INH had no strong effect on 103+ multiplication (Fig. 5B). In contrast, triclosan, an inhibitor of both FAS-I and FAS-II enoyl-CoA reductases (Liu *et al*., 2002), inhibited growth of both *M. smegmatis* and *R. equi* at a low concentration of 2 μg ml^−1^ (Fig. 5C), demonstrating that whereas FAS-II is dispensable only for *R. equi*, both bacteria required a functional FAS-I system.

### Virulence-related phenotypes of the *kasA* mutant

**Fig 6:**
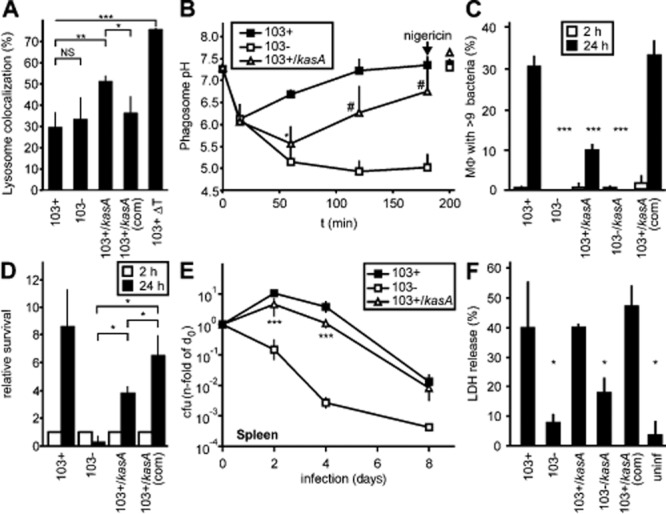
Pathogenicity-related phenotypes of the *kasA* mutant.A. Phagolysosome formation. Colocalization of intra-J774E macrophage bacteria with lysosomal BSA rhodamine was quantified using confocal laser scanning microscopy. Data are the means and standard deviations from four independent experiments. *P ≤ 0.05; **P ≤ 0.01; ***P ≤ 0.005.B. Development of phagosome pH during 180 min of infection, recorded using a ratiometric fluorescence determination with carboxyfluorescein as a fluor whose emission varies with pH versus rhodamine as a largely pH-independent emitting fluor. Nigericin (a K^+^/H^+^ antiporter) was added at 180 min of infection to collapse all pH gradients and to test the calibration of the system, emissions were measured 20 min later. Means and standard deviations from three independent experiments are shown. *P* is ≤ 0.05 only for 103+/*kasA* compared with 103+ at 60 min (*), and *P* ≤ 0.05 for 103+/*kasA* compared to 103− at 120 and 180 min (#).C. Multiplication in J774E macrophages was determined microscopically at 2 and 24 h of infection using the fluorescent DNA dye SYTO13. Macrophages with 10 or more bacteria were calculated as percentages of all infected macrophages. This quantification mode is necessary due to the intracellular clumping of *R. equi* (Hondalus and Mosser, 1994). Data are the means and standard deviations from three independent experiments.D. Experiments as in C but live bacteria counts were determined on nutrient agar from samples taken at the indicated times. Bacterial live cell counts in the 2 h samples are set as ‘1’ for each analysed strain.E. Virulence testing in mice. Approximately 5 × 10^5^ *R. equi* were administered intravenously per mouse and bacterial live cell counts determined at the indicated times of infection. Numbers of bacteria in the spleens were determined for each period of infection and sample and were normalized with each strain for the number of bacteria present at 2 h after infection (‘0 days’; see *Experimental procedures*). Data are the means and standard deviations of two independent infection experiments with four mice per time of infection and sample type in each experiment, except for 8 days infection experiments which were performed once with groups of four mice per infection period and sample type.F. Cytotoxicity of infection for J774E macrophages. Release of cytoplasmic lactate dehydrogenase (LDH) from macrophages was taken as a measure for plasma membrane rupture. Extracellular LDH was determined at 24 h of infection and compared with total macrophage LDH set as 100%. Means and standard deviations from three independent experiments are shown. *P ≤ 0.05; NS, not significant; ‘uninf’, uninfected; ‘103+/*kasA*(com)’, 103+/*kasA*, genetically complemented with the *R. equi KasA* wild-type gene, ‘103+ ΔT’, heat-killed *R. equi* 103+.

The *kasA* mutant had been isolated in a screen designed to isolate *R. equi* that are more frequently delivered to phagolysosomes. To test whether this was specifically true for 103+/*kasA*, phagolysosome formation was quantified with BSA rhodamine-preloaded lysosomes using confocal laser scanning microscopy of J774E murine macrophages. At 2 h of infection, the extent of phagolysosome formation was approximately the same for 103+ and 103−, in agreement with previous observations (Fernandez-Mora *et al*., 2005). The 103+/*kasA* mutants were, however, found significantly more frequently in phagolysosomes and this effect could be reversed by expressing the recombinant wild-type *kasA* gene in the mutant (Fig. 6A). The extent to which phagosome trafficking was normalized in mutant-containing phagosomes was similar to that seen with an *M. marinum kasB* mutant (5% phagolysosomal for the wild-type versus 35% for the mutant; Gao *et al*., 2003) or with various *M. tuberculosis* mutants which had been isolated using a similar phagolysosome enrichment approach (Pethe *et al*., 2004).

Acidification of phagosomes occurs upstream of phagosome–lysosome fusion (Haas, 2007). We quantified the kinetics of the average phagosome pH by ratiometric fluorescence analysis. At 1 h of infection, the average pH of phagosomes containing 103+ was 6.7, that of phagosomes containing 103− was 5.1 and that of 103+/*kasA* was 5.5 (Fig. 6B). The initial drop in pH of phagosomes containing 103+, followed by a rebound, is a typical pattern of phagosomes containing 103+ (von Bargen *et al*., 2011) and likely reflects an intraphagosomal activity of rhodococcal virulence-associated protein A (VapA) (Jain *et al*., 2003; von Bargen *et al*., 2009). 103+/*kasA* phagosomes share this initial pH drop but then their pH declines further until, after approximately 1 h of infection, alkalinization begins (Fig. 6B). This pattern was likely the reflection of an initially enhanced phagolysosome formation which led to incorporation of lysosomal vacuolar ATPase into the *R. equi* vacuoles, but, in this hypothesis, even many of these phagosomes would neutralize over time by VapA activity. In this scenario, those phagosomes which contained bacteria that had been heavily damaged by lysosome hydrolases do not return to neutral pH so that, at 3 h of infection, the average phagosome pH of mutant phagosomes is ∼ 0.5 units lower than in the corresponding 103+ samples (Fig. 6B).

Generally, exposure of intracellular pathogens to macrophage lysosome contents and acid is expected to lead to growth delay or even killing. To test whether the above increase in phagolysosome formation had this effect, intracellular multiplication of 103+ and of 103+/*kasA* in J774E cells was quantified both microscopically (Fig. 6C) and through determination of bacterial colony forming units (Fig. 6D). Using either mode of quantification, 103+/*kasA* bacteria multiplied significantly less in macrophages than 103+. Similar results were obtained when primary bone marrow-derived macrophages from C57BL/6 mice were used as host cells (not shown). It should be mentioned that uptake of 103+/*kasA* by macrophages was not significantly different from uptake of wild-type bacteria. Genetically complemented 103+/*kasA* bacteria multiplied normally in macrophages (Fig. 6C and D). Virulence was tested in a clearance model of intravenously infected C57BL/6 mice (Pei *et al*., 2006). Live bacterial counts in homogenized spleens were determined by plating on nutrient agar at 2 h, 2 days, 4 days and 8 days of infection. In spite of an initial increase in bacterial burden typical for mouse infection with virulent *R. equi*, bacterial counts were 40–60% lower for 103+/*kasA* than for 103+ bacteria at 2 days, 4 days and 8 days (Fig. 6E). This agrees well with the decreased multiplication observed in isolated macrophages (Fig. 6C and D) and could possibly be explained by the fact that approximately 50% of the bacteria had at least some contact with lysosome contents early and, hence, could not multiply. Full functional complementation of the *kasA* mutation by recombinant full-length *kasA* was observed at 4 days of infection (Fig. S3), a point in time when the differences between virulent and avirulent strains are maximal (Fig. 6E).

Enhanced cytotoxicity for murine J774E macrophages, a characteristic of virulent *R. equi*, was similar for infection with wild-type or the corresponding *kasA* mutant bacteria (Fig. 6F), suggesting that the *kasA* mutation did not affect this process. The mechanism of cytotoxic effects of *R. equi* is still enigmatic, yet requires neither expression of VapA nor intracellular multiplication of bacteria (von Bargen *et al*., 2009).

### Coating *E. coli* or 103*+*/*kasA* cells with long-chain mycolic acid compounds reduces phagolysosome formation

**Fig 7:**
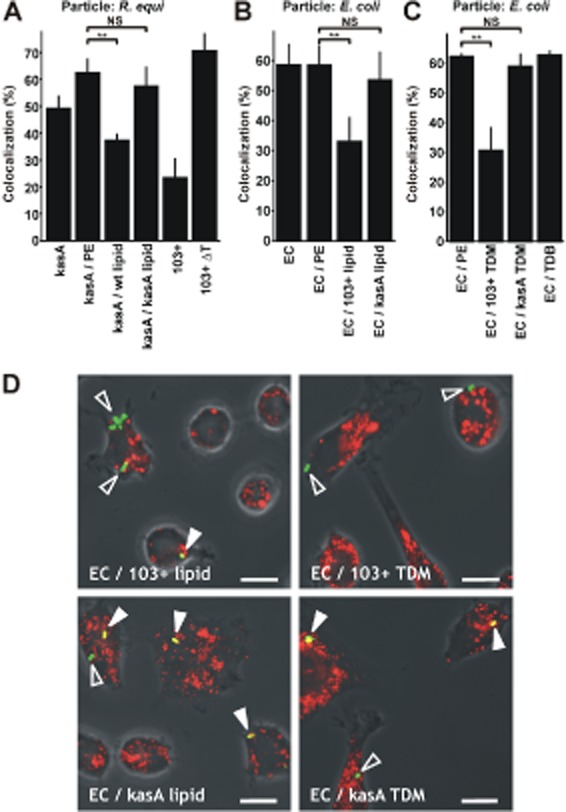
The roles of lipids in delivery to phagolysosomes. Lipids from *R. equi* 103+ or 103+/*kasA* were attached to *E. coli* DH5α or 103+/*kasA* cells. The fractions of intracellular green fluorescently labelled bacteria that were positive for lysosomal rhodamine (in case of *R. equi*) or fluorescent 20 nm nanobeads (in case of *E. coli*) was determined using confocal laser scanning microscopy. Data obtained with either labelling method are similar, yet we preferred nanobeads in later experiments due to their high fluorescence yield.A. 103+ lipids but not 103+/*kasA* lipids inhibit lysosome fusion of 103+/*kasA*-containing phagosomes. 103+, 103+/*kasA* (kasA), heat-killed 103+ (103+ ΔT) and 103+/*kasA* treated with the vehicle petrolether only (kasA / PE) or with PE containing 103+ wild-type lipids (kasA / wt lipid) or 103+/*kasA* lipids (kasA / kasA lipid) were used to infect J774E cells. Infection was for 20 min, followed by 20 min incubation in bacteria-free medium (chase). Phagolysosome formation was quantified by confocal laser scanning microscopy.B. 103+ lipids but not 103+/*kasA* lipids reduce lysosome fusion with *E. coli*-containing phagosomes. Infection was with untreated *E. coli* DH5α (EC), PE-treated (EC / PE) *E. coli* or *E. coli* coated with lipids extracted from 103+ (EC / 103+ lipid) or from 103+/*kasA* (EC / kasA lipid), each in PE. Infection was for 30 min, followed by a 30 min chase. Phagolysosome formation was quantified as above.C. 103+ TDM but not 103+/*kasA* TDM reduce lysosome fusion of *E. coli*-containing phagosomes. *E. coli* DH5α was coated with equal quantities of either TDM purified from 103+ (EC / 103+ TDM) or 103+/*kasA* (EC / kasA TDM) or with TDB (EC / TDB) or they were treated with petroleum ether only (EC / PE). Phagolysosome formation was quantified as above after 30 min of infection plus 30 min chase.D. Representative confocal laser scanning micrographs from *E. coli* samples as used in B and C. Overlays of green and red channels and transmitted light are shown. Open arrowheads, not fused with lysosomes; closed arrowheads, phagolysosomes. Bars, 10 μm.Data in A–C are the means and standard deviations of three independent experiments with at least 100 phagosomes counted for each sample and experiment. *P ≤ 0.05; **P ≤ 0.01; ***P ≤ 0.005.

The above data suggested that *R. equi* surface lipids participate in the manipulation of phagosome trafficking. To analyse whether lipid compounds play a direct role in diverting phagosome maturation, we used a non-pathogenic microorganism which does not naturally possess mycolic acids, which does not interfere with phagolysosome formation and which is readily killed by mouse macrophages (Dykstra *et al*., 2011), *Escherichia coli* strain DH5α. Lipid extracts from 103+ or 103+/*kasA* bacteria were used to coat *R. equi kasA* mutant (Fig. 7A) or *E. coli* (Fig. 7B), similarly as previously performed with purified lipids from mycobacteria and inert beads (Indrigo *et al*., 2003; Axelrod *et al*., 2008). Treatment with 103+ lipids significantly reduced phagosome–lysosome fusion whereas *kasA* mutant lipids had little, if any, effect, suggesting that long-chain mycolic acids were a traffic-diverting activity of 103+. As 103+ and 103+/*kasA* bacteria shared meromycolic acid chains up to 36 carbon units in length, but only wild-type bacteria possessed chains longer than that, this is likely the minimal length for lipid compounds that divert phagosome trafficking.

In a next step, we replaced the complex coating lipid mixture by a purified mycolic acid-containing compound, trehalose 6,6′-dimycolate (TDM). TDM is a major released mycolic acid compound from mycobacteria (Rhoades *et al*., 2003; Geisel *et al*., 2005) and a potent immunostimulant (Matsunaga and Moody, 2009). This makes it a prime candidate for a mycolic acid-containing effector molecule. TDM was purified from 103+ and 103+/*kasA* lipid extracts. Thin layer chromatography and mass spectrometric analysis confirmed that the obtained TDM was pure and structurally identical to the TDM analysed in crude chloroform : methanol extracts from complete cells (Fig. S4). TDM was used at 40 μg (3 × 10^7^)^−1^ cells to coat *E. coli* DH5α, and approximately 0.3 μg of either wild-type or mutant TDM associated with the coated bacteria (Fig. S5), demonstrating similar degree of coverage. *E. coli* coated with 103+ TDM was approximately 50% less frequently delivered to lysosomes than mock (petroleum ether)-treated *E. coli* whereas coating with 103+/*kasA* TDM reduced the frequency of phagolysosome formation very little (Fig. 7C and D). Trehalose 6,6′-dibehenate (TDB), a compound with one trehalose molecule and two saturated C_22_ alkane residues instead of mycolic acids at its 6 and 6′ positions, did not affect phagolysosome formation (Fig. 7C).

The mechanism by which long-chain mycolic acids interfere with phagolysosome formation remains to be elucidated. Mycobacterial TDM is the glycolipid which is most abundantly released from the bacterial surface (Geisel *et al*., 2005; Hunter *et al*., 2006). Some of it is likely incorporated into phagosome membranes (Spargo *et al*., 1991; Rhoades and Ullrich, 2000; Hayakawa *et al*., 2007; Astarie-Dequeker *et al*., 2009) where it would interact with phagosome lipids and proteins in a similar way to leishmanial lipophosphoglycan (Dermine *et al*., 2005). TDM may also alter the hydration status of the cytosolic face of the phagosome membrane (Harland *et al*., 2009) which is highly relevant to membrane fusion (Ge and Freed, 2011) or modulate the fate of *R. equi* by channelling them into host macrophages via specific receptors such as the recently identified TDM receptor, Mincle (Ishikawa *et al*., 2009; Schoenen *et al*., 2010). Mincle is, however, not likely directly involved in the observed effects, because it is a receptor for TDM as much as for TDB (Schoenen *et al*., 2010) and TDB did not have any trafficking diverting effects in our study. Finally, long-chain lipids could interdigitate between entire phagosome membrane leaflets, hence limiting fusion, signalling and formation of membrane lipid clusters, and in this way inhibit phagolysosome formation (Spargo *et al*., 1991; Crowe *et al*., 1994; Almog and Mannella, 1996). Because the 103+/*kasA* mutant TDM is shorter than 103+ TDM, it may be less efficient at altering such membrane characteristics.

## Conclusion

This study demonstrates a direct role of mycolic acid chain length in the manipulation of phagosome trafficking, although the effects of small decreases in very-long-chain mycolic acid chain length have previously been described for an *M. marinum kasB* mutant (Gao *et al*., 2003). This is the first report about an actinomycete whose mycolic chain length can be permanently and dramatically reduced without any strong effect for viability under our laboratory conditions. This observation explains the insensitivity of *R. equi* to antibiotics that are targeted against FAS-II components. However, shorter-chain mutants were partly attenuated in their virulence, allowing the conclusion that long-chain TDM can be a virulence factor and, at the same time, a dispensable housekeeping factor. In addition to short-term fusion inhibition by TDM and possibly similar lipids, virulence plasmid-encoded VapA is required to sustain the inhibition of phagolysosome formation, possibly by collapsing proton gradients across endocytic organelles (von Bargen *et al*., 2009). Further studies into the precise mechanisms of fusion inhibition by only long-chain TDM will likely reveal general principles of pathogenesis by virulent members of the mycobacteria–rhodococci–nocardiae group.

## Experimental procedures

### Chemicals, cells and infections

Chemicals used in this study were from Sigma-Aldrich (Taufkirchen, Germany) unless stated otherwise. Bacterial and mammalian cells, their cultivation and propagation and use in infection experiments are described in *Supporting information*.

### Bacterial strains and propagation

*Rhodococcus equi* 103+ was originally isolated from a pneumonic foal (Takai *et al*., 2000) and grown in BHI (BD Biosciences, Heidelberg, Germany) broth at 30°C (routine cultivation, stabilizes virulence plasmid) or 37°C (for experiments and expression of virulence-related genes) on a rotatory shaker at 200 r.p.m. or on BHI nutrient agar. 103− is the isogenic plasmid-less derivative (Fernandez-Mora *et al*., 2005). The chromosome and the VAP of 103+ have been sequenced (Takai *et al*., 2000; Letek *et al*., 2010). To cure *R. equi* of the VAP, the bacteria were grown at 37°C in broth for 2–3 weeks with subculturing every 2 days. Possible loss of the VAP in single clones was monitored regularly by immunodot blot using a monoclonal virulence-associated protein A (VapA) antibody (10G5; Takai *et al*., 1993). Where indicated, *R. equi* were grown in synthetic minimal media, composed of the minimal media base as in Ashour and Hondalus (2003) supplemented with a trace metal mixture as reported by Vishniac and Santer (1957). Minimal media contained 20 mM Na acetate as sole carbon source. Cultures were shaken at 30°C or 37°C, as indicated. *E. coli* XL-1Blue [F′::Tn10 *proA*+B+*lacI*q Δ(*lacZ*)M15/*recA1 endA*1 *gyrA*96 (Nalr) *thi hsdR*17(rK– mK+) *glnV*44 *relA*1 lac] and *E. coli* DH5α {F′/*endA*1 *hsdR*17 (rK–mK+) *glnV*44 *thi*-1 *recA*1 *gyrA* (Nalr) *relA*1 Δ(*lacI*ZYA*argF*) U169 *deoR* [φ80dlacΔ(lacZ)M15]} cells were propagated in Luria–Bertani (LB; Roth, Karlsruhe, Germany) agar at 37°C or in broth at 37°C and 190 r.p.m. In cloning experiments, antibiotics were used as follows: kanamycin at 50 μg ml^−1^ (*E. coli*) or 200 μg ml^−1^ (*R. equi*), and hygromycin (Invitrogen, Carlsbad, Germany) at 50 μg ml^−1^ (*E. coli*) or 100 μg ml^−1^ (*R. equi*). *M. smegmatis* mc^2^ 155 was kindly supplied by Georg Plum (University of Cologne, Germany) and grown on LB agar.

### Generation of *R. equi* transposon mutants and DNA manipulation

Transposon mutagenesis of *R. equi* 103+ using the EZ::TN <KAN-2> Tnp Transposome Kit (Epicentre Biotechnologies, Hess, Germany) was as described (Sydor *et al*., 2008) and 1000 individual mutant clones were pooled. Identification of the transposon insertion site by restricting chromosomal DNA with EcoRI and cloning it in *E. coli* using the EcoRI-digested Bluescript II (SK+) vector followed by selection for transposon-mediated kanamycin resistance was as in Sydor *et al*. (2008). A *kasA* construct for functional complementation of the mutant was produced by PCR amplification of the 1381 bp *kasA* gene from chromosomal DNA of strain 103+ using the primer pair AAACTGCAGCCACCAAGAACGACGAC (forward primer) and AAAAAGCTTGGTCATGATGTCCTCCTGAGC (reverse primer) and cloning into the EcoRI site of *E. coli*–*Mycobacterium* shuttle expression vector pSMT3 which provides a mycobacterial *hsp60* transcription promoter (Herrmann *et al*., 1996). Plasmid transfer into *R. equi* was by electroporation as in Sekizaki *et al*. (1998) followed by hygromycin selection.

### Magnetic enrichment of *R. equi*-containing phagolysosomes

To isolate *R. equi* mutants defective in inhibiting phagolysosome formation, a published protocol (Pethe *et al*., 2004) was modified by using 10 nm magnetic nanobeads as a lysosome tracer instead of iron dextran. The above pool of 1000 *R. equi* mutant types was grown from a −80°C stock for 4 h in 20 ml of BHI/kanamycin at 37°C on a rotatory shaker, harvested and resuspended in phosphate-buffered saline (PBS) by 10 passages through a 25-gauge syringe. To load lysosomes with the nanomagnets, three 10 cm plastic dishes with 5 × 10^6^ murine J774E macrophage-like cells each were incubated for 1 h with 5 ml of complete DMEM containing BSA-coated magnetic particles to load cells with particles [nanobeads were prepared as follows. Six microlitres of ferrofluid solution (EMG 508, Ferrotec, Unterensingen, Germany) was mixed with 1 ml of PBS containing 10 mg of bovine serum albumin (BSA) and incubated for 12 h on a tumbler at RT. The magnetic particles were separated by placing the tube in a magnetic holder (Dynal Biotech, Hamburg, Germany) for 30 min at ambient temperature with gentle tumbling. Medium was removed; the BSA-coated magnetic particles were resuspended in 6 ml of complete DMEM and sterile filtered]. To chase the magnetic particles that had been ingested for 1 h into lysosomes, macrophages were washed four times with warm PBS and incubated for 2 h. This is ample time for all particles to reach a compartment enriched in LAMP-1, mature cathepsin D and high lysosomal β-glucuronidase activity (Becken *et al*., 2010). Cells on each plate were infected with the above mutant pool of 1000 different *R. equi* transposon mutants for 1 h at 10 bacteria per macrophage, washed thrice with warm PBS and the infection was chased for 2 h in 5 ml of complete DMEM. Medium was replaced with 5 ml of homogenization buffer (HB, 250 mM sucrose/0.5 mM EGTA/0.1% gelatin/20 mM HEPES/KOH, pH 7.2) and cells were scraped off the plate with a rubber policeman. The cell suspension was transferred into a 15 ml Falcon tube and pelleted by 5 min centrifugation in a Hettich Universal 320R centrifuge at 160 *g* and 4°C. The supernatant was removed and cells were resuspended in 1 ml of chilled HB. Macrophages were lysed by 7 to 12 passages through a 27-gauge syringe. A post-nuclear supernatant was prepared by centrifugation at 250 *g* at 4°C for 10 min, and this was applied to a MiniMACS column (Miltenyi, Bergisch Gladbach, Germany) in its magnetic holder. The column was washed five times with 4 ml of cold HB each. Bound material was eluted in 1 ml of 0.5% Tween-20 in H_2_O, bacteria in the elution fraction were collected in a minifuge at 5220 *g* for 5 min, resuspended in deionized water and 100 μl portions were spread onto BHI/kanamycin agar plates. The whole screening procedure was repeated six times using the last selected pool of mutants in each enrichment.

### Macrophages, intracellular multiplication and mouse infection

The murine macrophage-like cell line J774E (Fernandez-Mora *et al*., 2005) was from Dr Philip D. Stahl (Washington University, St. Louis, USA) and was cultivated in ‘complete Dulbecco’s modified Eagles medium’ (DMEM; Invitrogen, Carlsbad, Germany) containing 10% fetal calf serum (FCS; from PAA, Pasching, Austria) and 1% Glutamaxx (Invitrogen) at 37°C and 7% CO_2_. Cells were split 1:3 every 2 to 3 days. Murine primary bone marrow-derived macrophages (BMMs) were generated (Sydor *et al*., 2008). Infection and microscopic quantification of intra-macrophage bacterial numbers were as described (Sydor *et al*., 2008) in a way that is customary in the *R. equi* field due to the fact that *R. equi* tends to stick to each other within phagosomes and is hard to disperse which makes plating on nutrient agar difficult (Hondalus and Mosser, 1994). However, quantification of live intracellular bacteria was (additionally) performed by nutrient agar plating of lysed infected macrophages (von Bargen *et al*., 2011). C57BL/6 mice were infected with approximately 5 × 10^5^ cfu in 0.3 ml of PBS per animal (Pei *et al*., 2006). The actual live cell count was determined subsequently from the inocula on BHI agar and was determined to be 2.7–4.2 × 10^5^ cfu. To adjust for these minor differences in the initial bacterial load and to determine how many bacteria were really within the mice, mouse spleens were removed 2 h after infection of the bacteria (*t*_0_) and homogenized in PBS. Ten-fold serial dilutions of the homogenates were plated on LB agar. The bacterial loads in spleens after 2, 4 and 8 days of infection were expressed as *x*-fold the bacterial load at *t*_0_ to normalize between infection experiments. The differences between cfu from spleens from infected mice of the same sample type at 2 h of infection did not exceed 20%. Experiments involving animals were approved by the University of Guelph Animal Care Committee (permit AUP_05R069) under the guidelines of the Canadian Committee on Animal Care.

### Analysis of virulence characteristics

Microscopic analysis of phagolysosome formation was performed as follows: J774E macrophages were seeded on glass coverslips as above. Complete DMEM containing 1.04 × 10^11^ red-fluorescent latex beads (diameter 20 nm) per millilitre or 50 μg ml^−1^ BSA rhodamine was added and cells incubated 16 h at 37°C and 7% CO_2_ in a humid atmosphere. Cells were rinsed with PBS twice to remove excess fluid marker and incubated in label-free complete DMEM for 2 h at 37°C/7% CO_2_ to chase the beads or BSA rhodamine into lysosomes (Schramm *et al*., 2008; Becken *et al*., 2010). Cells were infected with fluorescently labelled bacteria at 20 bacteria per macrophage for 20 min (*R. equi*) or at 30 particles for 30 min at 37°C/7% CO_2_ (*E. coli* DH5α). Non-phagocytosed bacteria were removed by rinsing twice with warm PBS and cells were incubated in fresh medium for 20 min (*R. equi*) or 30 min (*E. coli*) before fixation in 3% paraformaldehyde/PBS for 30 min at ambient temperature and quenching with 50 mM NH_4_Cl in PBS for 30 min. Samples were prepared for fluorescence microscopy by staining non-phagocytosed particles with rabbit *α*-DH5α antibody (1:50) or rabbit anti-*R. equi* serotype 6 antibody (1:200; C. Lämmler, Giessen, Germany), secondary antibody Cy5 goat anti-rabbit (1:200; Dianova, Hamburg, Germany). All antibodies were diluted in 5% BSA in PBS. Percentages of bacteria colocalizing with the fluorescent tracers were determined using a confocal laser scanning microscope (Zeiss LSM 510, Oberkochen, Germany). Average phagosome pH was determined by ratiometric fluorescence analysis of phagosomes that contained bacteria that had been covalently surface-labelled with fluorescein (pH-sensitive) and rhodamine (pH-insensitive) as described (Sydor *et al*., 2008). Cytotoxicity of the infection was quantified by release of cytoplasmic lactate dehydrogenase as described (Sydor *et al*., 2008) using the Cytotoxicity Detection Kit (Roche, Mannheim, Germany).

### Growth tests in various media, with or without antibiotics or lipid synthesis inhibitors

Plate assay to probe sensitivity to antibiotics: *R. equi* 103+ and 103+/*kasA* were grown overnight at 30°C at 190 r.p.m. in BHI broth and for each test plate 10^8^ bacteria were pelleted, resuspended in 100 μl of PBS and spread on a LB agar plate. Sterile Whatman filter paper (Dassel, Germany) disks (5 mm diameter) were soaked each with 10 μl of antibiotics solution (1 mg ml^−1^) in BHI broth. Agar plates and disks were briefly dried and, for each antibiotic and strain, six disks were placed evenly spaced onto the agar plates. After incubation at 30°C for 16 h, zones of complete growth inhibition were determined including the paper disks and the disk diameter (5 mm) was subtracted from each halo. Determination of minimum inhibitory concentration (MIC) in a 96 round-well format: serial dilutions of antibiotics were produced in BHI broth covering concentration range from 5 mg ml^−1^ to 2.44 μg ml^−1^. *R. equi* 103+ or 103+/*kasA* grown as above in BHI for 18 h were added per well at 1.5 × 10^5^ bacteria and the cultures in a total volume of 200 μl per well were left standing in a humidified incubator at 30°C for 16 h, centrifuged at 1200 *g* for 15 min. MIC was defined as the minimal antibiotic concentration at which bacteria did not form any detectable pellet.

For testing the effects of FAS inhibitors on bacterial growth, stationary 16 h 37°C cultures of *R. equi* in BHI or of *M. smegmatis* in LB broth were used to inoculate 3 ml of the respective media in glass test tubes with 0.001 OD_600_ equivalents of bacteria. Media were supplemented with isoniazide (Sigma Aldrich Biochemicals, catalogue number I3377), triclosan (offered as irgasan; Sigma Aldrich Biochemicals, Number 72779) or thiolactomycin sulfate (kindly supplied by R. E. Lee, Memphis, TN, USA) at the indicated concentrations from stocks in dimethyl sulfoxide. Incubation was in a rotatory shaker at 200 r.p.m. at 37°C for the indicated times at which 200 μl of samples were removed and OD_600_ determined. To control for possible solvent effects dimethyl sulfoxide was added at the highest concentration used in parallel tubes.

### Transmission *electron* microscopic analysis

Transmission *electron* microscopic analysis was as described (Sydor *et al*., 2008) but no ruthenium red was added.

### Extraction of bacterial lipids and lipid attachment

*Rhodococcus equi* was grown at 37°C to a late log phase under vigorous shaking (OD_600_ approximately 1.0, 50 ml of broth). Cultures were autoclaved at 121°C for 20 min and cells harvested by centrifugation at 1780 *g*, pellets resuspended in 5 ml of PBS and suspensions transferred into thoroughly washed screw glass cap centrifuge tubes. Cells were collected as above and lipids extracted by vortexing in 3 ml of chloroform : methanol (CM, 2:1), followed by 5 min water bath sonication at ambient temperature. Samples were stirred for 1 h with a magnetic stir bar. Cells were collected as above, supernatants were kept and 3 ml of CM (1:2) was added to each pellet. Extraction was repeated as above, followed by pooling each pair of supernatants and re-centrifugation to eliminate any possibly remaining bacteria. The extract was transferred into a pre-weighed glass tube, solvent was evaporated under a constant stream of nitrogen in a water bath at 50°C and lipid weight was determined. Samples were stored airtight at −20°C. To coat bacteria with these lipids, extracts were dissolved in petroleum ether (Sigma-Aldrich, Hamburg, 261734) at 2–8 mg ml^−1^ (TDM: 40 μg in 50 μl), and 50 μl of lipid solutions corresponding to lipids from equal numbers of cells or 50 μl of petroleum ether as vehicle control were added to 3 × 10^7^ bacteria. Bacteria were resuspended by pipetting up and down for 1 min plus vortex shaking for 1 min. Solvent was largely removed under a stream of nitrogen and bacteria were resuspended in 0.1 M NaHCO_3_ buffer (pH 8.3). Bacteria were fluorescently labelled by adding 50 μg ml^−1^ ATTO 488-NHS-Ester (Atto Tec, Siegen, Germany) and incubation for 30 min on ice in the dark. Bacteria were washed once with 20 mM Tris/HCl (pH 8.0) and twice with PBS and collected by centrifugation. Coating with purified TDM (below) was carried out in the same way.

### Isolation of TDM

For isolation of pure TDM, CM extracts of strains 103+ and 103+/*kasA* produced as above were separated on a column (7 × 1 cm) of silicagel 60 (Merck, Germany). The sample was dissolved and applied in CM 95:5 (v/v), and the column was eluted with the same solvent with successively applied 4 × 20 ml portions of each, CM 93:7 and CM 1:1 (all v/v). All obtained fractions were analysed by TLC on aluminium HPTLC silicagel 60 plates (Merck, Germany), developed with CM 18:4 (v/v) and stained with Mostain at 150°C. In case of strain 103+/*kasA*, the first 20 ml portion of CM 97:3 (v/v) contained pure TDM; however, the other three portions needed to be re-chromatographed (as above), and yielded additional pure TDM in the CM 97:3 (v/v) eluates. In case of strain 103+, the first three portions of CM 97:3 (v/v) contained pure TDM.

### TLC analysis of coated *E. coli* cells

Lipid extracts (CM 1:2, v/v) from 3 × 10^7^ *E. coli* cells coated in 40 μg of purified TDM were analysed by TLC on aluminium HPTLC silicagel 60 plates (Merck, Germany), developed with CM 18:4 (v/v) and stained with Mostain (1% w/v ceric sulfate and 2.5% w/v ammonium molybdate in 10% v/v aqueous sulfuric acid) at 150°C.

### Growth tests in various media, with or without antibiotics or lipid synthesis inhibitors

Growth tests in various media, with or without antibiotics or lipid synthesis inhibitors were carried out by growing bacteria in rich BHI broth or minimal media with acetate on a rotatory shaker and recording the optical density at 600 nm at the indicated times as described in *Supporting information*. Growth inhibition tests were carried out by spotting 10 μg of antibiotic on a sterile filter paper and placing it on nutrient agar onto which bacteria have been plated. Inhibition zones were measured after incubation as described in *Supporting information*.

### Mass spectrometry analysis of rhodococcal lipids

Mass spectrometry analysis of rhodococcal lipids was performed by tandem mass spectrometry with electrospray ionization as described (Hsu *et al*., 2011a,b2011b) using chloroform methanol (1:2) extracts of rhodococci (above) when complex extracts were used. Purified TDMs were analysed by matrix-assisted laser desorption/ionization Fourier-transform mass spectrometry (MALDI FT-MS) in the positive ion mode using an APEX-Qe instrument (Bruker Daltonics, Billerica, USA). Matrix solution was prepared from 2,5-dihydroxybenzoic acid (2,5 DHB) in 1% trifluoroacetic acid at a concentration of 15 μg μl^−1^. Matrix and sample solution (0.2 μg μl^−1^) were mixed (1:1 v/v) and applied onto the stainless steel sample plate as 1.5 μl of droplets.

### Statistics

Data are expressed as means ± standard deviations. Significance of differences was analysed by two-tailed unpaired Student’s *t*-test.

## Notes

Fig. S1 Amino acid sequence comparison of KasA from *R. equi* with KasA enzymes from different actinomycetes. Amino acid sequences are aligned for *R. equi* KasA (GenBank accession number JN_030359) *Rhodococcus jostii* RHA1 (YP_701185), *Rhodococcus erythropolis* (BA_E66720), *Nocardia farcinica* IFM 10152 (YP_117827), *Mycobacterium avium* ssp. *paratuberculosis* K-10 (NP_960932), and *Mycobacterium tuberculosis* JH37Rv (NP_216761). Black (grey) background indicates amino acid residues which are identical (homologous) in the listed proteins.

Fig. S2 Sensitivity of 103+ and 103+/*kasA* to antibiotics. A. Sensitivity of 103+ and 103+/*kasA* to 16 antibiotics was tested in an agar diffusion test. The zone of growth inhibition is indicated in mm from the perimeter with 10 μg antibiotic was applied per disk. An expected pronounced cross-resistance to neomycin as a consequence of the transposon’s kanamycin resistance marker gene served as internal control. B. Minimal inhibitory concentrations were determined for 5 antibiotics selected for their increased effect on 103+/*kasA* from (A) in a broth dilution experiment. All data are presented as means and standard deviations from 3 independent experiments. *, *P* ≤ 0.05.

Fig. S3 Functional complementation of the 103+/*kasA* multiplication deficiency in mice by the wild type *kasA* gene. Approximately 5 × 10^5^ *R. equi* were administered intravenously bacteria per mouse and bacterial live cell counts determined at 2 h of infection (‘d’) or 4 d of infection. Numbers of CFU were normalized for each strain for the number of bacteria present at 0 d. Data are the means and standard deviations of two to four independent infection experiments with 4 mice per time of infection and sample type in each experiment. Please note that the icons for 103+ and complemented mutant (103+/KasA complem.) overlap at 4 d of infection. Means and standard deviations are shown. **, significance of difference from 103+ calculates as *P* < 0.01.

Fig. S4 Mass spectrometric analysis of purified TDM used in coating experiments. Purified TDM from 103+/*kasA* (top) and 103+ (bottom) were analysed in the positive ion mode by MALDI FT-MS. The mass regions between *m/z* 500 and 1720 are plotted against relative signal intensity. The essential lack of low molecular weight compounds demonstrates the purity of the TDM preparations.

Fig. S5 TDM isolated from 103+ or 103+/*kasA* attach equally well to *E. coli*. *E. coli* DH5α were coated with TDM purified from 103+ (A) or 103+/*kasA* (B) and extracted using chloroform : methanol (2:1, then 1:2), following the extraction protocol for TDM from *R. equi*. Extracts were analysed by thin layer chromatography. TDM I and TDM II represent extractions from two different coated *E. coli* samples. 0.1 and 0.5 μg of purified TDM were added as standards in (A) and (B). The arrow indicates the migration position of TDM (which is missing in the uncoated mock samples), stars indicate the running positions of co-extracted *E. coli* lipids.
