# Supplementary figures and images for: Diversion of phagosome trafficking by pathogenic Rhodococcus equi depends on mycolic acid chain length
Source: Cell Microbiol. 2012 Nov 13;15(3):458–73. doi: 10.1111/cmi.12050 (PMC3864644; doi:10.1111/cmi.12050)

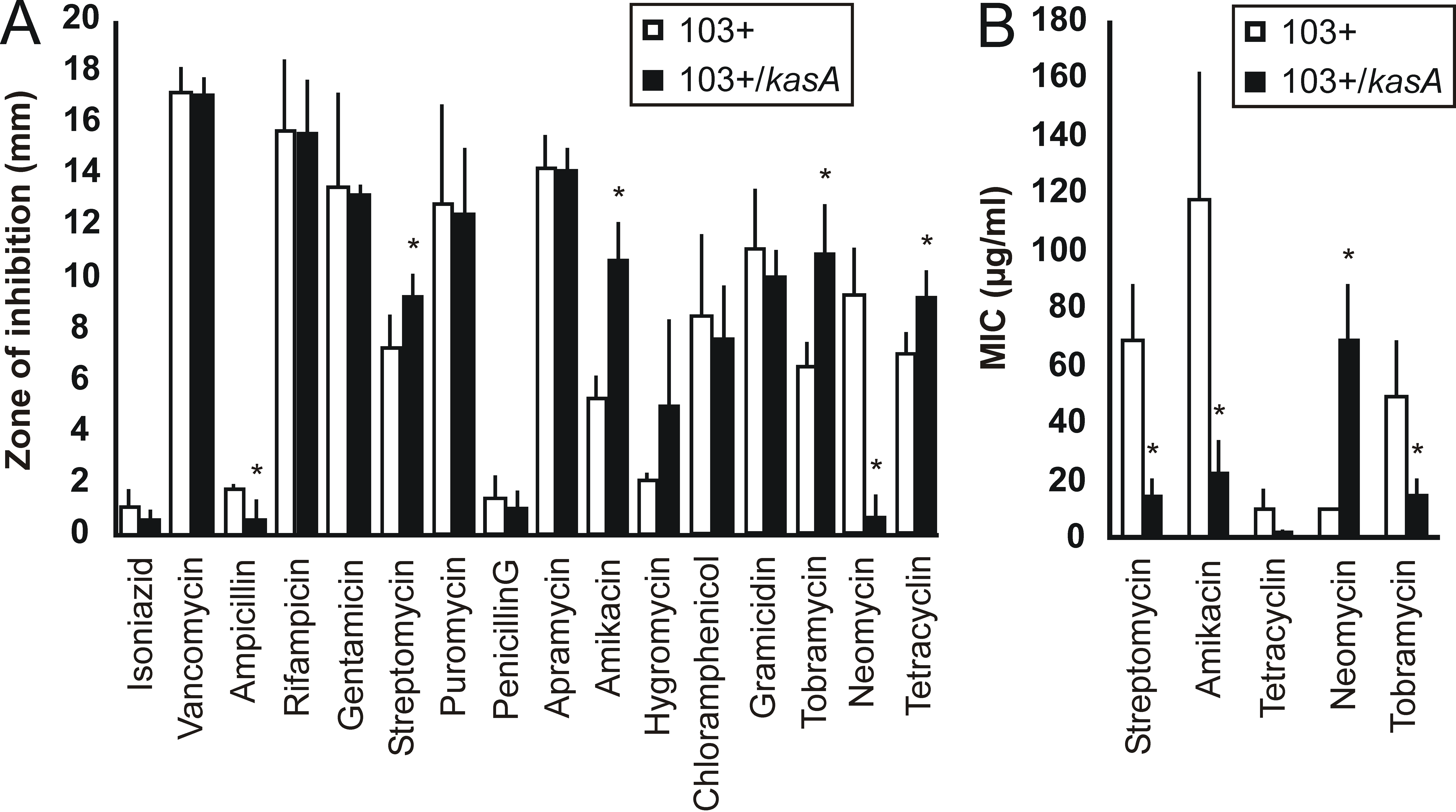

Supplement: Fig. S2 — Sensitivity of 103+ and 103+/kasA to antibiotics. A. Sensitivity of 103+ and 103+/kasA to 16 antibiotics was tested in an agar diffusion test. The zone of growth inhibition is indicated in mm from the perimeter with 10 μg antibiotic was applied per disk. An expected pronounced cross-resistance to neomycin as a consequence of the transposon’s kanamycin resistance marker gene served as internal control. B. Minimal inhibitory concentrations were determined for 5 antibiotics selected for their increased effect on 103+/kasA from (A) in a broth dilution experiment. All data are presented as means and standard deviations from 3 independent experiments. *, P ≤ 0.05. [file cmi0015-0458-sd2.tif]

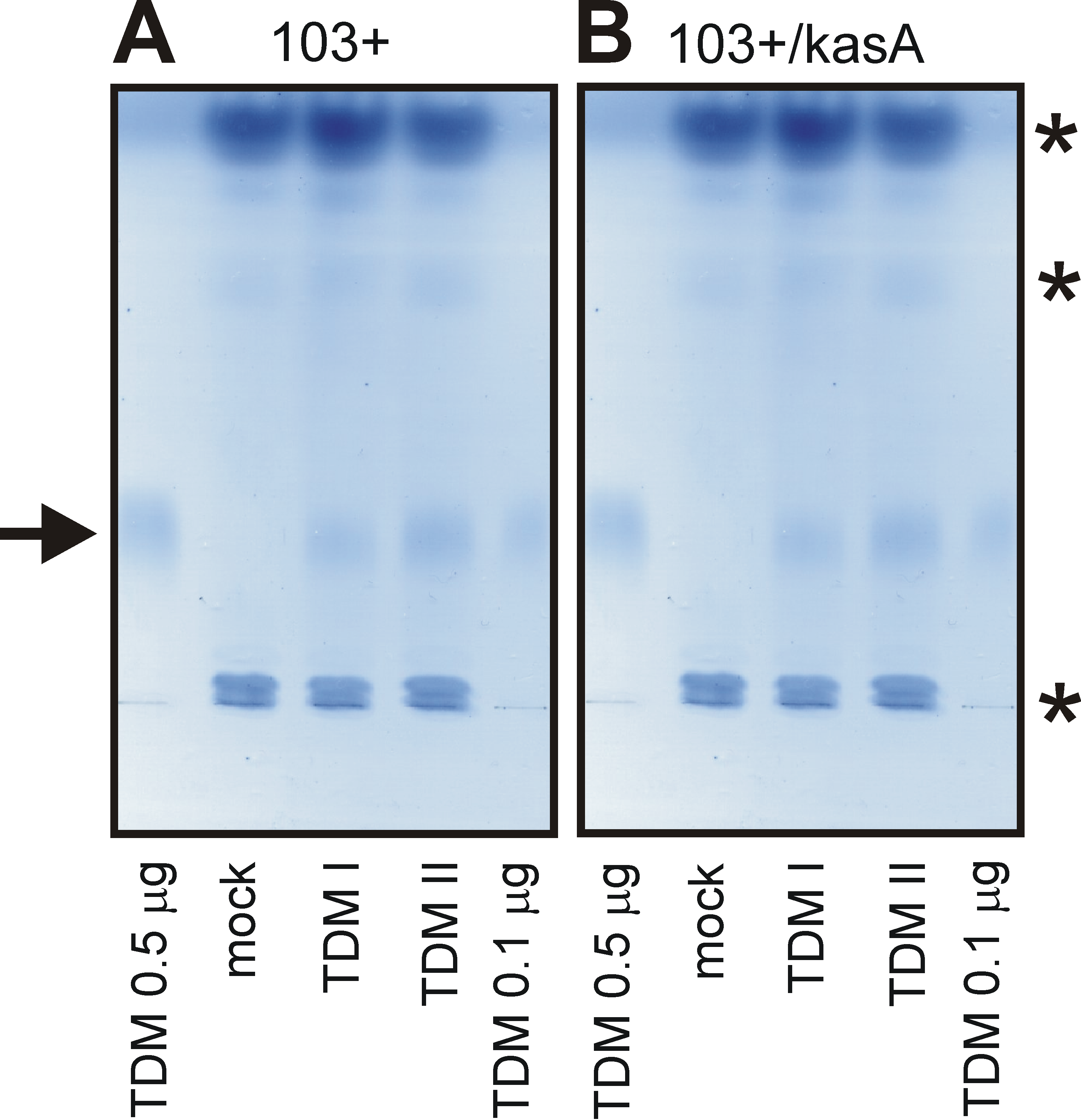

Supplement: Fig. S4 — Mass spectrometric analysis of purified TDM used in coating experiments. Purified TDM from 103+/kasA (top) and 103+ (bottom) were analysed in the positive ion mode by MALDI FT-MS. The mass regions between m/z 500 and 1720 are plotted against relative signal intensity. The essential lack of low molecular weight compounds demonstrates the purity of the TDM preparations. [file cmi0015-0458-sd4.tif]

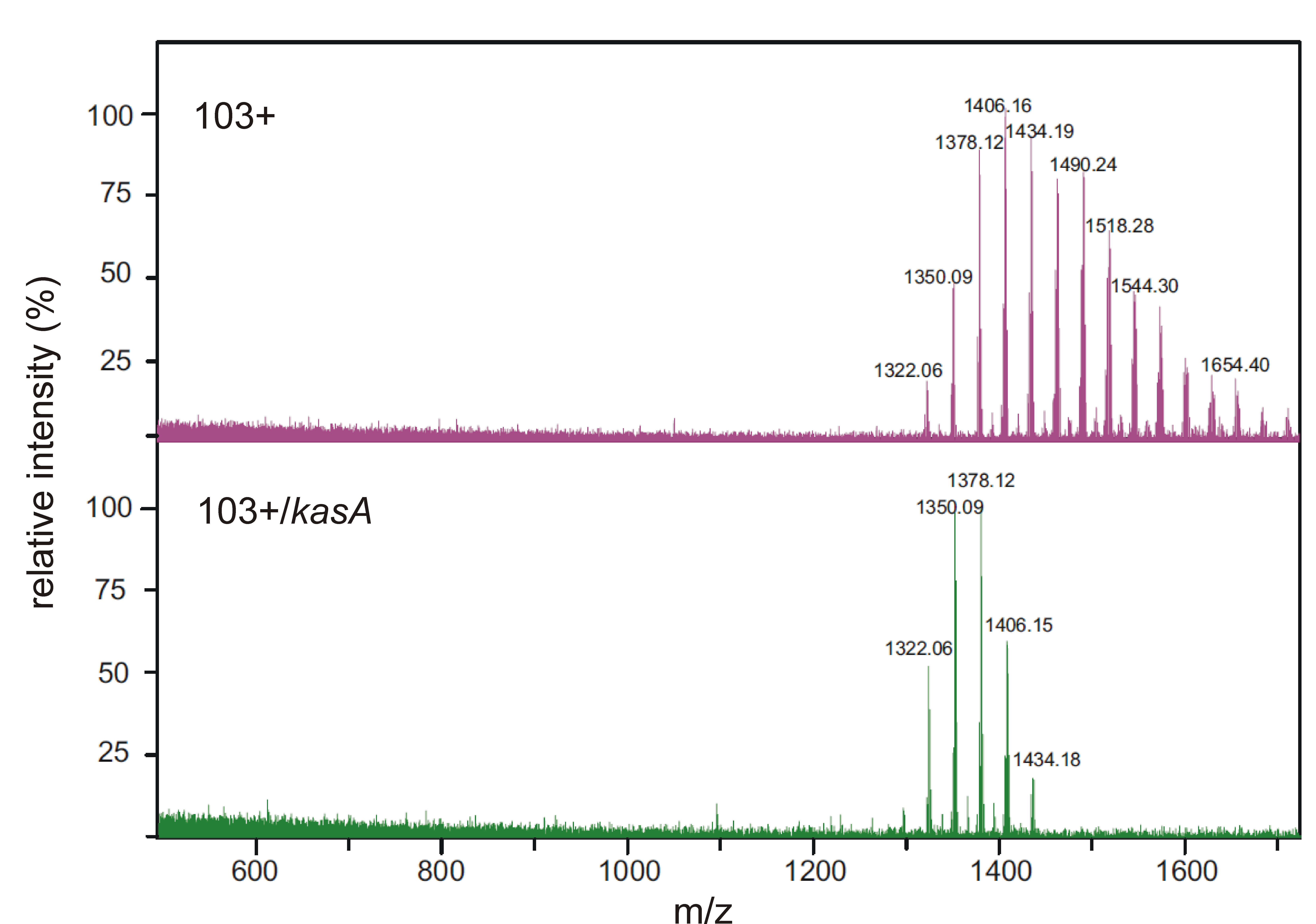

Supplement: Fig. S5 — TDM isolated from 103+ or 103+/kasA attach equally well to E. coli. E. coli DH5α were coated with TDM purified from 103+ (A) or 103+/kasA (B) and extracted using chloroform : methanol (2:1, then 1:2), following the extraction protocol for TDM from R. equi. Extracts were analysed by thin layer chromatography. TDM I and TDM II represent extractions from two different coated E. coli samples. 0.1 and 0.5 μg of purified TDM were added as standards in (A) and (B). The arrow indicates the migration position of TDM (which is missing in the uncoated mock samples), stars indicate the running positions of co-extracted E. coli lipids. [file cmi0015-0458-sd5.tif]
