# Supplementary material for: Diversion of phagosome trafficking by pathogenic Rhodococcus equi depends on mycolic acid chain length
Source: Cell Microbiol. 2012 Nov 13;15(3):458–73. doi: 10.1111/cmi.12050 (PMC3864644; doi:10.1111/cmi.12050)
Supplement: Supplementary file 6 [file cmi0015-0458-sd6.doc]

**Fig. S1.**Amino acid sequence comparison of KasA from *R. equi* with KasA enzymes from different actinomycetes. Amino acid sequences are aligned for *R. equi* KasA (GenBank accession number JN_030359) *Rhodococcus jostii* RHA1 (YP_701185), *Rhodococcus erythropolis* (BA_E66720), *Nocardia farcinica* IFM 10152 (YP_117827), *Mycobacterium avium* ssp. *paratuberculosis* K-10 (NP_960932), and *Mycobacterium tuberculosis* JH37Rv (NP_216761). Black (grey) background indicates amino acid residues which are identical (homologous) in the listed proteins.

**Fig. S2.**Sensitivity of 103+ and 103+/*kasA* to antibiotics.

A. Sensitivity of 103+ and 103+/*kasA* to 16 antibiotics was tested in an agar diffusion test. The zone of growth inhibition is indicated in mm from the perimeter with 10 µg antibiotic was applied per disk. An expected pronounced cross-resistance to neomycin as a consequence of the transposon’s kanamycin resistance marker gene served as internal control.

B. Minimal inhibitory concentrations were determined for 5 antibiotics selected for their increased effect on 103+/*kasA* from (A) in a broth dilution experiment. All data are presented as means and standard deviations from 3 independent experiments. *, *P* ≤ 0.05.

**Fig. S3.**Functional complementation of the 103+/*kasA* multiplication deficiency in mice by the wild type *kasA* gene. Approximately 5 × 10^5^ *R. equi* were administered intravenously bacteria per mouse and bacterial live cell counts determined at 2 h of infection (‘d’) or 4 d of infection. Numbers of CFU were normalized for each strain for the number of bacteria present at 0 d. Data are the means and standard deviations of two to four independent infection experiments with 4 mice per time of infection and sample type in each experiment. Please note that the icons for 103+ and complemented mutant (103+/KasA complem.) overlap at 4 d of infection. Means and standard deviations are shown. **, significance of difference from 103+ calculates as *P* < 0.01.

**Fig. S4.**Mass spectrometric analysis of purified TDM used in coating experiments. Purified TDM from 103+/*kasA* (top) and 103+ (bottom) were analysed in the positive ion mode by MALDI FT-MS. The mass regions between *m/z* 500 and 1720 are plotted against relative signal intensity. The essential lack of low molecular weight compounds demonstrates the purity of the TDM preparations.

**Fig. S5.**TDM isolated from 103+ or 103+/*kasA* attach equally well to *E. coli*. *E. coli* DH5α were coated with TDM purified from 103+ (A) or 103+/*kasA* (B) and extracted using chloroform : methanol (2:1, then 1:2), following the extraction protocol for TDM from *R. equi*. Extracts were analysed by thin layer chromatography. TDM I and TDM II represent extractions from two different coated *E. coli* samples. 0.1 and 0.5 μg of purified TDM were added as standards in (A) and (B). The arrow indicates the migration position of TDM (which is missing in the uncoated mock samples), stars indicate the running positions of co-extracted *E. coli* lipids.
